# Systematic Review and Meta-Analysis of the Application of T-PEP in the Therapeutic Management of COPD Patients

**DOI:** 10.3390/jcm14020320

**Published:** 2025-01-07

**Authors:** Arianna Sepiacci, Nadia Starc, Rossella Laitano, Franco Pasqua, Paola Rogliani, Josuel Ora

**Affiliations:** 1 Unit of Respiratory Medicine, Department of Experimental Medicine, University of Rome “Tor Vergata”, 00133 Rome, Italy; 2 Pulmonary Rehabilitation Unit, Clinical Rehabilitation Institute of IRCCS San Raffaele, 00163 Rome, Italy; 3 Division of Respiratory Medicine, University Hospital Tor Vergata, 00133 Rome, Italy

**Keywords:** chronic obstructive pulmonary disease, chronic mucus hypersecretion, airway clearance techniques, temporary positive expiratory pressure, pulmonary rehabilitation

## Abstract

**Background:** Chronic obstructive pulmonary disease (COPD) is a leading cause of morbidity and mortality worldwide, characterized by chronic mucus hypersecretion (CMH) that exacerbates airway obstruction and accelerates disease progression. Effective airway clearance techniques are essential to improve respiratory function and reduce exacerbations. Temporary Positive Expiratory Pressure (T-PEP) is a novel airway clearance device that has shown promise in managing COPD. **Objectives**: This meta-analysis aimed to evaluate the efficacy of T-PEP in a standard pulmonary rehabilitation program. **Methods**: Following PRISMA guidelines, a comprehensive search of randomized controlled trials (RCTs) was conducted in the MEDLINE and PEDro databases. Data from 162 subjects, including those with severe COPD and bronchiectasis, were analyzed. Key outcomes assessed were changes in lung function (FVC, FEV1, TLC), inspiratory and expiratory pressures (MIP, MEP), gas exchange (PaO_2_, PaCO_2_), exercise capacity (6MWT), symptom severity (mMRC, CAT, BCSS), and exacerbation rates. **Results**: T-PEP significantly improved FVC, FEV1, TLC, MIP, MEP, and DLCO compared to baseline, with heterogeneity noted across studies. Improvements in gas exchange and physical capacity were observed, with PaO_2_ increasing and PaCO_2_ decreasing. T-PEP also reduced symptoms of cough and dyspnea, improving quality-of-life scores. Additionally, a notable reduction in acute exacerbations of COPD was seen after one month and three months of treatment. **Conclusions**: T-PEP therapy shows substantial benefits in improving lung function, exercise capacity, and quality of life while reducing exacerbation rates in COPD patients. Although promising, these findings require further confirmation through randomized clinical trials to establish the optimal application of T-PEP in various clinical settings and patient phenotypes.

## 1. Introduction

Chronic obstructive pulmonary disease (COPD) is a preventable and treatable condition characterized by persistent respiratory symptoms and airflow limitation due to structural abnormalities in the airways and alveoli. These abnormalities typically arise from prolonged exposure to harmful particles or gases, such as those from cigarette smoke or environmental pollutants. The 2024 Global Initiative for Chronic Obstructive Lung Disease (GOLD) [1] classifies COPD as a leading cause of morbidity and mortality worldwide [2], with acute exacerbations playing a critical role in the accelerated decline in lung function [3]. This condition not only severely impacts patients’ quality of life (QoL) but also imposes a significant financial burden on healthcare systems.

A key pathological feature of COPD is chronic mucus hypersecretion (CMH), which is strongly associated with worsening lung function, increased frequency of exacerbations, higher rates of hospitalization, and elevated mortality [4]. Exposure to irritants such as tobacco smoke induces submucosal gland hypertrophy and goblet cell metaplasia, driving excessive mucus production via complex signaling pathways [5,6]. Patients with CMH experience more severe bacterial airway colonization and more frequent and intense exacerbations compared to those without CMH [7]. These changes lead to a marked decline in health status and lung function, underscoring the need for effective management strategies targeting mucus clearance. While both pharmacological and non-pharmacological treatments exist, optimizing treatment approaches remains a priority [8].

Pulmonary rehabilitation (PR) is a cornerstone of COPD management, offering significant improvements in exercise tolerance, symptom control, and health-related quality of life [9,10]. PR is particularly effective in addressing the mucus hypersecretion that exacerbates airway obstruction and accelerates disease progression. Among the rehabilitation techniques, airway clearance strategies play a critical role in optimizing lung function and reducing exacerbation rates [11]. These techniques, such as positive expiratory pressure (PEP) devices, are designed to mobilize mucus, improve ventilation, and relieve airflow limitations [12].

Temporary Positive Expiratory Pressure (T-PEP) is an advanced airway clearance device that applies a consistent low level of pressure during expiration, facilitating mucus removal and improving respiratory function [13]. T-PEP operates by shifting the equal pressure point peripherally, thereby enhancing mucus clearance and reducing dynamic lung hyperinflation [14]. Unlike oscillatory PEP (OPEP) devices, which use vibration to dislodge mucus, T-PEP provides a stable expiratory pressure, making it particularly advantageous for COPD patients with CMH [15,16].

Some clinical studies have investigated the benefits of T-PEP in COPD management, demonstrating its potential to reduce exacerbation frequency, improve lung function parameters (such as forced vital capacity [FVC] and forced expiratory volume in one second [FEV1]), and enhance patient-reported outcomes, including dyspnea and quality-of-life scores [15,16,17,18]. Although T-PEP is gaining traction in clinical practice, there remains a need for a thorough evaluation of its effectiveness across diverse clinical settings, such as in-hospital treatment and home care.

This meta-analysis aims to consolidate existing evidence regarding the therapeutic benefits of T-PEP in COPD management. By reviewing data from multiple studies, this analysis will evaluate the device’s impact on reducing exacerbations, improving lung function, and enhancing quality of life, thereby offering valuable insights into its role in pulmonary rehabilitation and informing future clinical research directions.

## 2. Materials and Methods

### 2.1. Search Strategy and Study Eligibility

**Figure 1:**
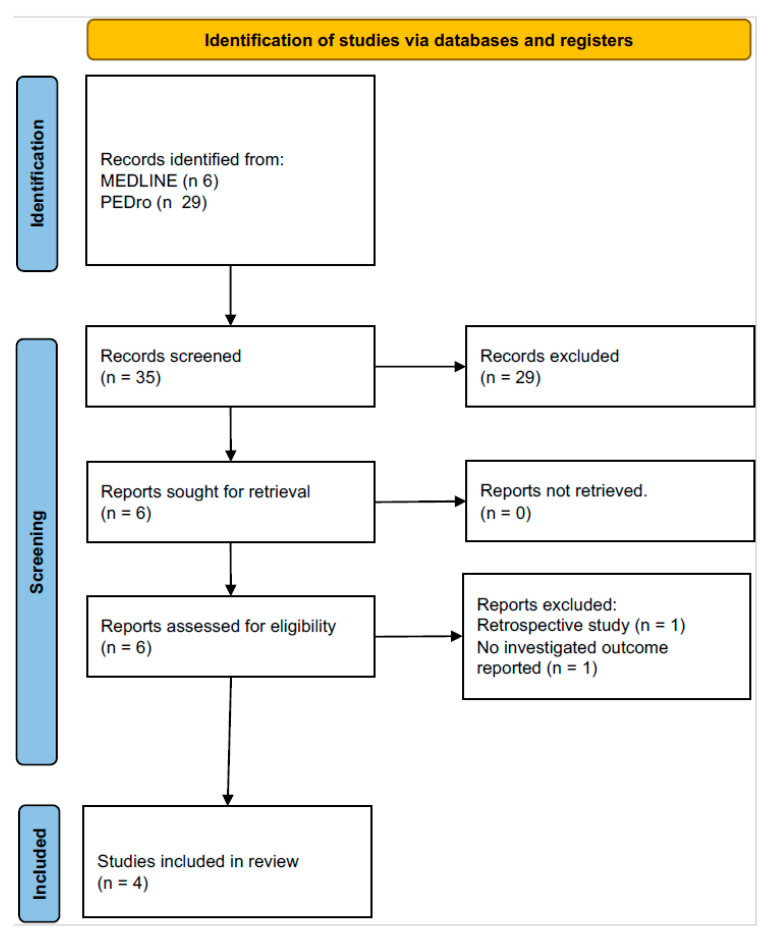
PRISMA flowchart. The diagram illustrates the identification, screening, eligibility, and inclusion process of studies analyzed and included in this meta-analysis.

This systematic review and meta-analysis was performed according to the PRISMA-P guidelines [19,20]. The PRISMA 2020 flow diagram is shown in Figure 1, and the PRISMA-P checklist is reported in Table S1. A comprehensive literature search was carried out for randomized controlled trials (RCTs) assessing TPEP in obstructive pulmonary diseases. The patient problem, intervention, comparison, and outcome (PICO) framework was applied for the literature search. The “patient problem” included obstructive pulmonary disease, the “intervention” focused on T-PEP, the “comparison” was TPEP vs. medical treatment or other different airway clearance techniques (ACTs), and the “outcome” included improvements in lung function, arterial blood gas (ABG), and health status assessment tests (Modified Medical Research Council Dyspnea Scale (mMRC); Breathlessness, Cough, and Sputum Scale (BCSS); COPD Assessment Test (CAT)), as well as exacerbation rates.

The search was conducted in the MEDLINE and PEDro databases to find relevant studies published up to 1 September 2024. The search string used was (T-PEP OR Temporary positive expiratory pressure) AND (obstructive pulmonary disease [MeSH Terms] OR asthma OR bronchiectasis OR bronchiolitis OR chronic obstructive pulmonary disease). This review was not registered and the protocol not prepared.

### 2.2. Study Selection

Only randomized controlled trials (RCTs) were included in the systematic review and meta-analysis. Studies were excluded if they did not report data on changes in respiratory function, quality of life, or exacerbation rates. Two reviewers independently performed the systematic article search, resolving discrepancies through discussion.

### 2.3. Data Collection

Data from the RCTs were extracted from the journal articles. All data related to respiratory function tests were sought, including absolute values or percentage predicted values; data on respiratory symptoms and quality of life using the following scales: mMRC, CAT, and BCSS; and, where possible, the number of exacerbations. The main data of the studies are summarized in Table 1, which shows the characteristics of each study, the duration, the number, the demographic characteristics of the patients analyzed, the type of TPEP intervention, the intervention performed in the control group, and the outcomes obtained.

### 2.4. Endpoint

The primary aim of this study was to evaluate the effectiveness of TPEP in the management of chronic obstructive respiratory diseases. The key outcomes assessed included improvements in respiratory function and quality of life, and a reduction in the frequency of exacerbations.

Outcomes were compared both before and after TPEP therapy, as well as between the TPEP treatment group and the control group, providing a comprehensive evaluation of the device’s therapeutic impact.

### 2.5. Data Synthesis and Analysis

The results were presented as mean ± SD for variation (Δ). The variation (Δ) was directly extracted from some articles, while in others, it was calculated by subtracting the pre-treatment value from the post-treatment value. When available, mean values were provided along with their standard deviations. When not explicitly provided, these values were estimated from the confidence interval using OpenMeta software (http://www.cebm.brown.edu/openmeta/download.html (accessed on 1 October 2024)).

A meta-analysis was conducted on lung function values (FVC%, FEV1%, TLC%, MIP, MEP, DLCO%), dyspnea and symptom scores (mMRC, CAT, BCSS), exercise tests (6 min walking test), blood gas values (pO_2_ and pCO_2_), and exacerbation rates (after 1 month and after 3 months), first among patients treated with TPEP and then compared with the control group.

The results were analyzed utilizing Open Meta [Analyst] software version 2. The Q-test and I^2^ index were employed to assess heterogeneity, with a significance level of heterogeneity set at *p* < 0.10. Heterogeneity was categorized as follows: I^2^ < 25% (no heterogeneity), I^2^ between 25% and 50% (moderate heterogeneity), I^2^ between 50% and 75% (large heterogeneity), and I^2^ > 75% (extreme heterogeneity).

## 3. Results

### 3.1. Study Characteristics

Out of the six eligible articles [14,15,16,17,18,21] identified through the search, four were selected and included in the study (see flowchart in Figure 1). De Macedo et al. [14] was excluded, as it was a review, and the Herrero-Cortina study [21] was excluded due to focusing primarily on mucus clearance, which differed from the outcomes of the other studies.

Among the selected studies [14,15,16,17,18,21], one [15] was considered as two distinct groups because it analyzed T-PEP usage in two different settings: hospital and home, both compared with the same control group. The selected articles were published between 2014 and 2018, with a total of 503 patients, 163 of whom underwent TPEP therapy. Three studies [15,16,17] included patients with severe COPD (FEV1 < 50%), while one study [18] focused on patients with COPD, chronic bronchitis, or bronchiectasis, with a peak cough expiratory flow > 150 L/min and sputum production > 30 mL/day. TPEP therapy was administered in sessions ranging from 15 to 30 min, twice daily, for 10–15 days. The study durations varied between 10 days and 26 weeks, with therapy conducted either in hospital settings or at home.

### 3.2. Dynamic Lung Volumes (FEV1 and FVC)

**Figure 2:**
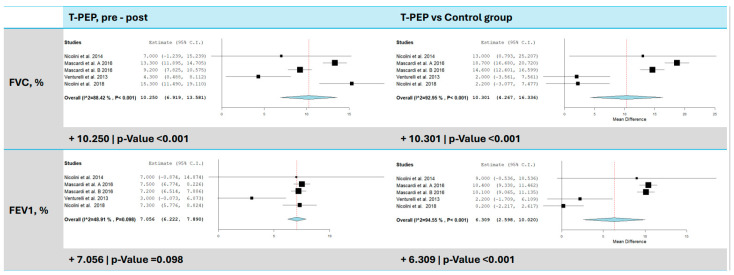
FEV1 and FVC comparison between T-PEP and control groups. The left panels display the comparison of values pre- and post-TPEP therapy, while the right panels show the comparison between the T-PEP group and the control group. FEV1 (forced expiratory volume in 1 s), FVC (forced vital capacity), T-PEP (Temporary Positive Expiratory Pressure) [15,16,17,18].

All studies [15,16,17,18] included in the meta-analysis evaluated changes in dynamic lung volumes, specifically FEV1 and FVC, measured as a percentage of predicted values (Figure 2). Four studies assessed changes in FVC following TPEP treatment, revealing a significant improvement, with an average increase of 10.25% compared to baseline values (95% CI: 6.92–13.58, *p* < 0.001). Heterogeneity was high (I^2^ = 88.42%, τ^2^ = 10.92, Q(df = 4) = 34.55, *p* < 0.001), indicating substantial variability across studies. When comparing the TPEP group to the control group, it was seen that FVC increased by 10.30% (95% CI: 4.27–16.34, *p* < 0.001), though heterogeneity remained high (I^2^ = 92.95%, τ^2^ = 39.08, Q(df = 4) = 56.70, *p* < 0.001). FEV1 also showed a significant improvement, with an average increase of 7.06% compared to baseline (95% CI: 6.22–7.89, *p* < 0.001, I^2^ = 48.91%, *p* = 0.098) and 6.31% compared to the control group (95% CI: 2.60–10.02, *p* < 0.001). Heterogeneity was very high (I^2^ = 94.55%, τ^2^ = 14.44, Q(df = 4) = 73.36, *p* < 0.001).

### 3.3. Total Lung Capacity (TLC)

**Figure 3:**
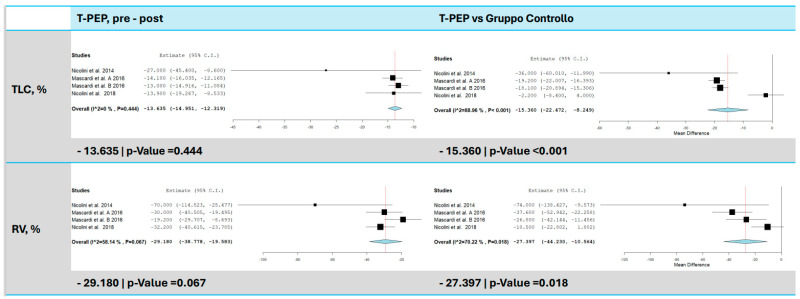
TLC and RV comparison between T-PEP and control groups. The left panels display the comparison of values pre- and post-TPEP therapy, while the right panels show the comparison between the T-PEP group and the control group. TLC (total lung capacity), RV (residual volume), T-PEP (Temporary Positive Expiratory Pressure) [15,16,17,18].

TLC was evaluated in three trials [15,16,17], with results indicating a significant reduction following TPEP therapy (Figure 3). On average, TLC decreased by 13.64% compared to baseline (95% CI: −14.95 to −12.32, *p* < 0.001). No heterogeneity was observed among the studies (I^2^ = 0%, τ^2^ = 0.00, Q(df = 3) = 2.680, *p* = 0.444), suggesting consistent results across studies. In comparison to the control group, TLC decreased by 15.36% (95% CI: −22.47 to −8.25, *p* < 0.001), although heterogeneity was high (I^2^ = 88.96%, τ^2^ = 38.08, Q(df = 3) = 27.18, *p* < 0.001).

### 3.4. Maximum Inspiratory Pressure (MIP) and Maximum Expiratory Pressure (MEP)

**Figure 4:**
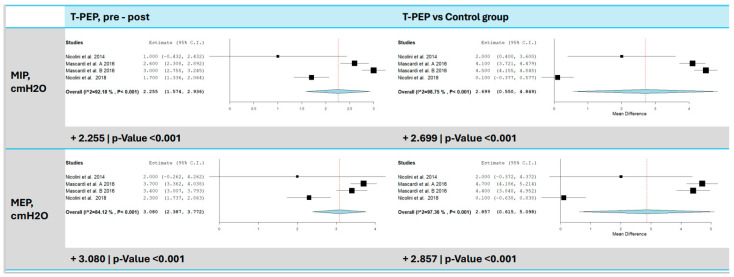
MIP and MEP comparison between T-PEP and control groups. The left panels display the comparison of values pre- and post-TPEP therapy, while the right panels show the comparison between the T-PEP group and the control group. MIP (maximum inspiratory pressure), MEP (maximum expiratory pressure), T-PEP (Temporary Positive Expiratory Pressure) [15,16,17,18].

Three studies [15,16,17] assessed changes in MIP following TPEP treatment, with a significant increase observed (Figure 4). The meta-analysis showed an average improvement of 2.26 cmH_2_O compared to baseline (95% CI: 1.57–2.94, *p* < 0.001) and 2.70 cmH_2_Ocompared to the control group (95% CI: 0.55–4.85, *p* = 0.014). Heterogeneity was high, indicating substantial variability across results. Similarly, MEP was evaluated in four trials, showing an average increase of 3.08 cmH_2_O compared to baseline (95% CI: 2.39–3.77, *p* < 0.001) and 2.86 cmH_2_O compared to the control group (95% CI: 0.62–5.10, *p* = 0.013).

### 3.5. Diffusing Capacity of the Lungs for Carbon Monoxide (DLCO%)

**Figure 5:**
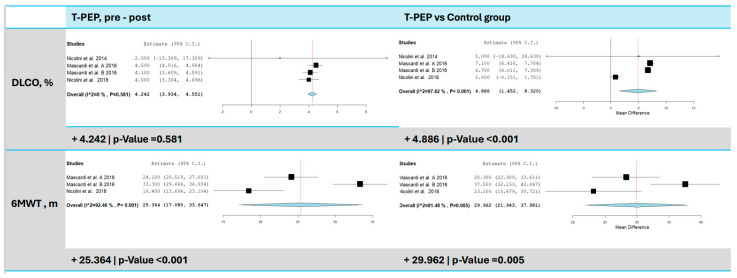
6MWT and DLCO comparison between T-PEP and control groups. The left panels display the comparison of values pre- and post-TPEP therapy, while the right panels show the comparison between the T-PEP group and the control group. 6MWT (Six-Minute Walk Test), DLCO (diffusing capacity of the lung for carbon monoxide), T-PEP (Temporary Positive Expiratory Pressure) [15,16,17,18].

DLCO was measured in three studies [15,16,17] (Figure 5), and TPEP treatment resulted in a significant increase in DLCO of 4.24% compared to baseline (95% CI: 3.93–4.55, *p* < 0.001). No heterogeneity was found among the studies (I^2^ = 0%, τ^2^ = 0.00, Q(df = 3) = 0.581, *p* = 0.901), suggesting consistency. However, when compared to the control group, the increase was 4.89% (95% CI: 1.45–8.32, *p* = 0.005), with high heterogeneity (I^2^ = 97.62%, τ^2^ = 9.31, Q(df = 3) = 126.11, *p* < 0.001).

### 3.6. Six-Minute Walk Test (6MWT)

Two trials [15,16] evaluated physical capacity using the 6MWT (Figure 5). The analysis revealed a significant improvement in distance walked, with an average increase of 25.36 m post-TPEP therapy (95% CI: 17.08–33.65 m, *p* < 0.001, I^2^ = 92.46%, *p* < 0.001) and an average increase of 29.96 m compared to the control group (95% CI: 21.94–37.98 m, *p* < 0.001, I^2^ = 81.48%, *p* = 0.005).

### 3.7. Blood Gas Analysis (pO_2_ and pCO_2_)

**Figure 6:**
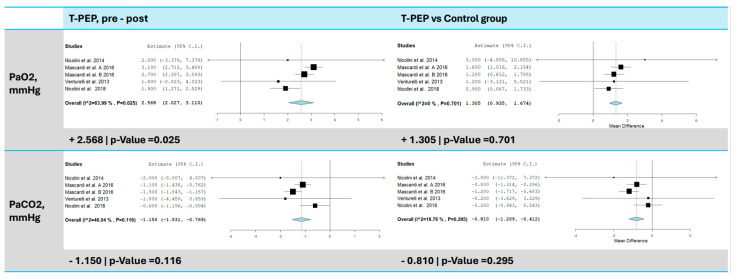
PaO_2_ and PaCO_2_ comparison between T-PEP and control groups. The left panels display the comparison of values pre- and post-TPEP therapy, while the right panels show the comparison between the T-PEP group and the control group. PaO_2_ (partial pressure of oxygen), PaCO_2_ (partial pressure of carbon dioxide), T-PEP (Temporary Positive Expiratory Pressure) [15,16,17,18].

All included studies [15,16,17,18] assessed changes in blood gases (pO_2_ and pCO_2_) following TPEP treatment (Figure 6). A significant increase in pO_2_ was observed, with an average improvement of 2.57 mmHg compared to baseline (95% CI: 2.03–3.11, *p* < 0.001, I^2^ = 63.99%, *p* = 0.025), and a significant reduction in pCO_2_, with an average decrease of 1.15 mmHg compared to baseline (95% CI: −1.53 to −0.77, *p* < 0.001, I^2^ = 46.04%, *p* = 0.116). Compared to the control group, pO_2_ increased by 1.31 mmHg (95% CI: 0.94–1.67, *p* < 0.001) and pCO_2_ decreased by 0.81 mmHg (95% CI: −1.21 to −0.41, *p* < 0.001).

### 3.8. Symptoms and Quality of Life (mMRC, CAT, BCSS)

**Figure 7:**
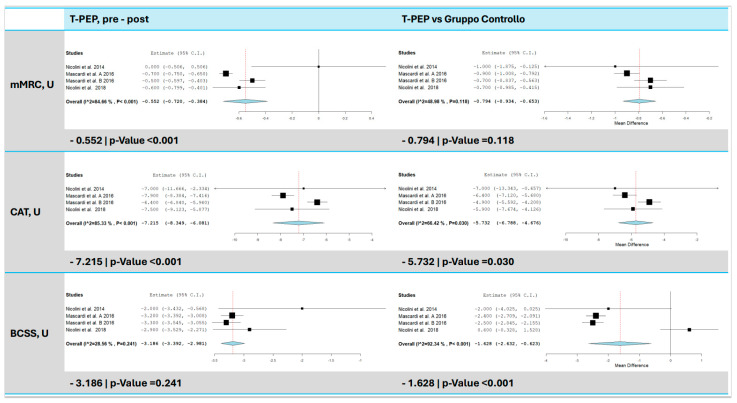
mMRC, CAT, and BCSS score comparison between T-PEP and control groups. The left panels display the comparison of values pre- and post-TPEP therapy, while the right panels show the comparison between the T-PEP group and the control group. mMRC (Modified Medical Research Council Dyspnea Scale), CAT (COPD Assessment Test), BCSS (Baseline Chronic Symptoms Scale), T-PEP (Temporary Positive Expiratory Pressure) [15,16,17,18].

TPEP treatment improved patients’ quality of life and reduced symptoms such as cough and dyspnea (Figure 7). Three studies [15,16,17] used the mMRC, CAT, and BCSS scales for assessment. The mMRC score decreased by 0.55 points (95% CI: −0.72 to −0.38, *p* < 0.001, I^2^ = 84.67%, *p* < 0.001) compared to baseline and by 0.79 points (95% CI: −0.93 to −0.65, *p* < 0.001, I^2^ = 48.98%, *p* = 0.118) compared to the control group. CAT scores dropped by 7.22 points (95% CI: −8.35 to −6.08, *p* < 0.001, I^2^ = 85.33%, *p* < 0.001) compared to baseline and by 5.73 points (95% CI: −6.79 to −4.68, *p* < 0.001, I^2^ = 66.42%, *p* = 0.030) compared to the control group. BCSS scores decreased by 3.19 points (95% CI: −3.39 to −2.98, *p* < 0.001, I^2^ = 28.56%, *p* = 0.241) compared to baseline and by 1.63 points (95% CI: −2.63 to −0.62, *p* = 0.001, I^2^ = 92.34%, *p* < 0.001) compared to the control group.

### 3.9. Acute Exacerbations of COPD (AECOPD)

**Figure 8:**
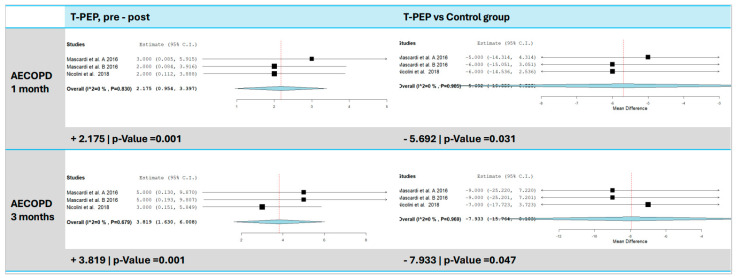
AECOPD episode comparison between T-PEP and control groups. The left panels display the comparison of AECOPD (acute exacerbations of COPD) episodes at 1 and 3 months pre- and post-TPEP therapy, while the right panels show the comparison between the T-PEP group and the control group. AECOPD (acute exacerbations of chronic obstructive pulmonary disease), T-PEP (Temporary Positive Expiratory Pressure) [15,16,17,18].

Two studies [15,16] were included to assess the impact of T-PEP on the number of acute exacerbations of COPD (AECOPD) after one month and after three months (Figure 8). Compared to baseline, the analysis revealed a significant reduction, with an average decrease of 2.18 events after 1 month (95% CI: 0.95–3.40, *p* < 0.001, I^2^ = 0%, *p* = 0.830) and an average decrease of 3.82 events after 3 months (95% CI: 1.63–6.01, *p* < 0.001, I^2^ = 0%, *p* = 0.679).

A significant reduction in AECOPD was also observed when compared to the control group. The analysis showed an average decrease of 5.69 events (95% CI: −10.86 to −0.53, *p* = 0.031, I^2^ = 0%, *p* = 0.985) after 1 month and an average decrease of 7.93 events after 3 months (95% CI: −15.76 to −0.10, *p* = 0.047, I^2^ = 0%, *p* = 0.969).

## 4. Discussion

This meta-analysis evaluated the efficacy of T-PEP in subjects with COPD and demonstrated a significant improvement in dynamic lung volumes, lung hyperinflation, respiratory muscle function, exercise tolerance, lung function, symptoms, and exacerbation frequency. These findings were consistent when comparing both pre- and post-treatment results within the treatment group, as well as when comparing the treated group to the control group.

There are several airway clearance techniques (ACTs) that use different strategies to eliminate excess secretions [11]. Their aim is to reduce airway obstruction caused by secretions occupying the airway lumen to prevent respiratory tract infections, re-expand the collapsed areas of the lung, improve gas exchanges, and decrease the inflammatory response. Among these ACTs, T-PEP is a compact, electrically powered device that operates during approximately two-thirds of the expiratory phase, allowing the patient to complete exhalation independently [22]. It is a noninvasive, gentle yet effective therapy that works with the naturally low pressures in the alveoli, aiding the airways in detaching and clearing secretions. T-PEP also produces a slight oscillation in the airflow, which facilitates secretion removal more effectively [11].

Although the results of this meta-analysis are highly positive for T-PEP, they must be interpreted within the context of the inclusion criteria, study duration, and therapeutic setting. The main limitations of the available clinical trials include their short duration, the severity of the patient population, and the predominantly hospital-based settings. Furthermore, the lack of comparisons with other devices makes it challenging to evaluate the cost–benefit ratio effectively.

All the studies included subjects with severe COPD, defined as having an FEV1 < 50% predicted, with the exception of the Venturelli et al. study [18], which enrolled stable patients (no exacerbation in the previous month) presenting with chronic mucus hypersecretion related to COPD or bronchiectasis. Nicolini et al. (2018) [16] and Mascardi et al. [15] focused on stable COPD patients who had not experienced exacerbations in the past two months, particularly those with chronic bronchitis without predominant bronchiectasis. Nicolini et al. (2014) [17] also included stable patients with severe COPD, although no specific details regarding phlegm production were provided. Despite some variations in the study populations, most patients had severe COPD with chronic bronchitis and were in a stable phase without recent exacerbations. These inclusion criteria inherently limit the application of T-PEP.

Our meta-analysis found significant improvements in dynamic lung volumes (FVC and FEV1), with T-PEP therapy showing an average increase of 10% in FVC and 7% in FEV1 compared to baseline. Similarly, a 14% reduction in TLC observed in our analysis further supports T-PEP’s efficacy in decreasing air trapping and improving lung mechanics. Additionally, our study demonstrated a statistically significant improvement in MIP and MEP of 2–3 cmH_2_O. Although these changes are not clinically significant, it is important to note that the reduction in TLC reflects a true deflation rather than inspiratory muscle weakness, underscoring the mechanical benefits of T-PEP therapy. These findings are partially consistent with the meta-analysis by De Macedo et al. [14], which did not demonstrate improvements in FEV1 and FVC but did confirm reductions in static lung volumes, including RV and TLC. In contrast, PEP masks showed limited efficacy in reducing hyperinflation, highlighting a potential advantage of T-PEP in this context.

For many of these studies, the therapy was conducted in a hospital or hospital-related setting (inpatient rehabilitation programs or outpatient clinics). Conversely, the study by Mascardi et al. [15] explored the differences in adherence and efficacy of T-PEP when administered at home versus in the hospital. All eligible patients, after randomization, received a one-hour training session in the lung laboratory with a physiotherapist or specialized nurse to ensure proper use and acclimatization to T-PEP before being included in the study protocol. Interestingly, there were no differences in outcomes or adherence between the two settings, though patients expressed a preference for the home setting. This study remains the only one to date to provide evidence for the domiciliary use of T-PEP, emphasizing the need for further research on its use outside the hospital.

Another point of relevance is the length of the therapy and the duration of the evaluation period. The longest trial, conducted by Nicolini et al. (2018), involved 12 days of therapy with a follow-up period of 26 weeks. In contrast, the other trials generally included therapy durations of 10–15 days with follow-up periods ranging from 10 days to 15 weeks. This highlights that, to date, all evidence supporting the use of T-PEP in COPD subjects is based on relatively short therapy durations, although the maximum benefits were observed in the longer trial [14,15]. Another challenge of pulmonary rehabilitation is the ability to maintain its long-term effects after completion [23]. However, to date, there are no available data on the long-term outcomes of T-PEP therapy.

Even with the limitations of the study duration, this meta-analysis interestingly demonstrated a reduction in acute exacerbations in COPD subjects with the use of T-PEP. Mucus dysfunction is a key factor in COPD, leading to the formation of mucus plugs that obstruct airways and impair gas exchange [24]. These plugs, observed in 25% to 67% of CT scans in COPD patients, are linked to airflow obstruction, hypoxia, and increased risk of infection, exacerbations, and pneumonia [5,24,25]. By facilitating the removal of these plugs, T-PEP may provide benefits beyond simple airway clearance. Based on this premise, the benefit of T-PEP may extend beyond simple airway clearance to facilitating the effective removal of these plugs. In light of this evidence, the observed improvement in gas exchange, reduction in exacerbations, and enhanced DLCO could hold a deeper and more significant clinical relevance, underscoring the potential role of T-PEP in targeting mucus dysfunction at its core.

The final point of discussion is the comparison between T-PEP and other airway clearance techniques (ACTs), which is crucial for evaluating both efficacy and cost-effectiveness. Nicolini et al. (2018) [16] compared T-PEP, OPEP, and a control group, demonstrating that while both devices improved dyspnea scales, lung function parameters, and health status, only T-PEP significantly reduced exacerbations after 1 and 3 months compared to the control group.

A 4-year retrospective study further compared the efficacy of T-PEP and PEP-mask in 162 subjects with COPD and bronchiectasis [26]. Both groups showed significant physiological improvements, with no major differences overall. However, T-PEP appeared to have specific advantages in improving gas transfer for patients with emphysema and those on oxygen therapy, whereas PEP showed greater efficacy in enhancing forced expiratory flow in mechanically ventilated patients. This study also highlighted that the therapy durations were relatively short, typically around 10 days, and conducted in hospital settings. These findings confirm the efficacy of T-PEP while underscoring the need for further research to determine if it is superior to other ACTs. Additionally, the reliance on short, hospital-based rehabilitation programs raises questions about the long-term applicability and cost-effectiveness of T-PEP in broader clinical contexts.

While the results of this meta-analysis are promising, several limitations should be noted. First, the heterogeneity observed in outcomes such as FVC and FEV1 indicates variability in patient populations and study methodologies, which may influence the consistency of the results. Furthermore, the small number of trials included in the analysis restricts the generalizability of the findings to broader COPD populations. Future studies should prioritize larger and more diverse cohorts to confirm the therapeutic impact of T-PEP across different COPD phenotypes.

Additionally, while T-PEP has shown efficacy in reducing exacerbations and improving lung function, there is a lack of data on its long-term effects, particularly regarding its role in preventing disease progression and maintaining improvements over time. Further research should also investigate head-to-head comparisons between T-PEP and other airway clearance techniques, such as oscillatory PEP and intermittent positive pressure breathing (IPPB), to better define its relative efficacy and cost-effectiveness in clinical practice.

There are several limitations to this meta-analysis. First, although we rigorously followed PRISMA guidelines, the absence of a previously registered protocol may reduce the transparency and reproducibility of the review. Second, only a few studies met our inclusion criteria, and most had small and relatively homogeneous sample sizes, which limits the generalizability of the findings; moreover, there are currently no additional studies in the literature, underscoring the need for further randomized controlled trials (RCTs) on this topic. Third, the short follow-up periods in these studies prevent a clear assessment of T-PEP’s long-term efficacy. Fourth, significant heterogeneity in lung function outcomes may affect the reliability of the conclusions. Finally, the lack of comparisons with other airway clearance techniques hinders a comprehensive evaluation of T-PEP’s relative effectiveness. Some of these limitations stem from the current paucity of research on T-PEP therapy, highlighting the urgency for well-designed, large-scale RCTs.

This meta-analysis highlights the potential efficacy of T-PEP therapy in improving various clinical parameters in COPD patients, including dynamic lung volumes, lung hyperinflation, respiratory muscle function, and exacerbation frequency. The observed improvements in gas exchange and reduction in TLC underscore the mechanical benefits of T-PEP in addressing air trapping and mucus dysfunction. Additionally, the findings suggest that T-PEP may offer specific advantages in certain subgroups, such as patients with emphysema or those on oxygen therapy.

Future research should prioritize larger, multicenter studies with longer therapy durations and follow-up periods to better understand the sustainability of T-PEP’s benefits. Additionally, randomized controlled trials comparing T-PEP with other ACTs could provide valuable insights into its relative efficacy and cost-effectiveness. Ultimately, these efforts will help refine the therapeutic role of T-PEP in COPD management and broaden its applicability in clinical practice.

## Figures and Tables

**Table 1:** Overview of included studies.

	Number Identifier	Study Characteristics	Main Inclusion Criteria	Setting	Therapy Time	Study Duration (Weeks)	Simple Size				Age Years (SD)			Male %		
							Total	IG	CG	AIG	IG	CG	AIG	IG	CG	AIG
Nicolini et al., 2014 [17]	Chi CTR-TRC-12002178	Single-blind randomized trial	40–80 years old, COPD (FEV1 < 50%)	Day hospital of the Respiratory Medicine Unit	30 min session twice a day, 5 days per week	3 weeks	45	15	15	15	73 (6.00)	70 (6.00)	70.00 (9.00)	60	53.3	60
Mascardi et al., 2016 a [15]	Chi-CTR-TRC 15006662	Randomized controlled study	COPD (FEV1 < 50%)	Hospital	30 min session twice a day for 15 days	15 weeks	120	35	35	0	71.7 (4.60)	70.7 (6.30)	0	71.8	71.8	0
Mascardi et al., 2016 b [15]	Chi-CTR-TRC 15006662	Randomized controlled study	COPD (FEV1 < 50%)	Hospital	30 min session twice a day for 15 days	15 weeks	120	34	35	0	70.7 (6.10)	70.7 (6.30)	0	75	71.8	0
Venturelli et al., 2013 [18]	NCT00700388	Single-blind multicenter randomized trial	COPD and/or chronic bronchitis or bronchiectasis with a peak cough expiratory flow > 150 l/min and sputum production > 30 mL/day	Inpatient pulmonary rehabilitation	15 min session twice a day for 10 days	10 days	98	44	39	0	70 (10.80)	71.6 (8.70)	0	54.7	77.8	0
Nicolini et al., 2018 [16]	Chi-CTR-IPR-16008487	Randomized controlled study	COPD (FEV1 < 50%), >35 anni	Outpatient Respiratory Unit	30 min session twice a day for 12 days	26 weeks	120	35	33	36	72.15 (1.20)	71.13 (1.90)	70.67 (2.10)	72.5	75	70

## Supplementary Materials

The following supporting information can be downloaded at: https://www.mdpi.com/article/10.3390/jcm14020320/s1, Table S1: PRISMA 2020 Checklist [27].

## List of Abbreviations

ABG	Arterial blood gas
ACTs	Airway clearance techniques
AECOPD	Acute exacerbations of COPD
BCSS	Breathlessness, Cough, and Sputum Scale
CAT	COPD Assessment Test
CMH	Chronic mucus hypersecretion
COPD	Chronic obstructive pulmonary disease
DLCO	Diffusing capacity of the lungs for carbon monoxide
FEV1	Forced expiratory volume in one second
FVC	Forced vital capacity
IPBB	Intermittent positive pressure breathing
MEP	Maximum expiratory pressure
mMRC	Modified Medical Research Council Dyspnea Scale
MIP	Maximum inspiratory pressure
OPEP	Oscillatory Positive Expiratory Pressure
pCO_2_	Partial pressure of carbon dioxide
PEP	Positive expiratory pressure
PICO	Patient, intervention, comparison, outcome
pO_2_	Partial pressure of oxygen
PR	Pulmonary rehabilitation
PRISMA-P	Preferred Reporting Items for Systematic Review and Meta-Analysis Protocols
QoL	Quality of life
RCTs	Randomized controlled trials
RV	Residual volume
6MWT	Six-Minute Walk Test
T-PEP	Temporary positive expiratory pressure
TLC	Total lung capacity
